# The diagnostic value of endoscopy and *Helicobacter pylori *tests for peptic ulcer patients in late post-treatment setting

**DOI:** 10.1186/1471-230X-4-27

**Published:** 2004-10-26

**Authors:** Heidi-Ingrid Maaroos, Helena Andreson, Krista Lõivukene, Pirje Hütt, Helgi Kolk, Ingrid Kull, Katrin Labotkin, Marika Mikelsaar

**Affiliations:** 1 Department of Family Medicine, Faculty of Medicine, University of Tartu, Estonia; 2 Department of Microbiology, Faculty of Medicine, University of Tartu, Estonia; 3 Department of Internal Medicine, Faculty of Medicine, University of Tartu, Estonia

## Abstract

**Background:**

Guidelines for management of peptic ulcer patients after the treatment are largely directed to detection of *H. pylori *infection using only non-invasive tests. We compared the diagnostic value of non-invasive and endoscopy based *H. pylori *tests in a late post-treatment setting.

**Methods:**

Altogether 34 patients with dyspeptic complaints were referred for gastroscopy 5 years after the treatment of peptic ulcer using a one-week triple therapy scheme. The endoscopic and histologic findings were evaluated according to the Sydney classification. Bacteriological, PCR and cytological investigations and ^13^*C-UBT *tests were performed.

**Results:**

Seventeen patients were defined *H. pylori *positive by ^13^*C-UBT *test, PCR and histological examination. On endoscopy, peptic ulcer persisted in 4 *H. pylori *positive cases. Among the 6 cases with erosions of the gastric mucosa, only two patients were *H. pylori *positive. Mucosal atrophy and intestinal metaplasia were revealed both in the *H. pylori *positive and *H. pylori *negative cases. Bacteriological examination revealed three clarithromycin resistant *H. pylori *strains. Cytology failed to prove validity for diagnosing *H. pylori *in a post-treatment setting.

**Conclusions:**

In a late post-treatment setting, patients with dyspepsia should not be monitored only by non-invasive investigation methods; it is also justified to use the classical histological evaluation of *H. pylori *colonisation, PCR and bacteriology as they have shown good concordance with ^13^*C-UBT*. Moreover, endoscopy and histological investigation of a gastric biopsy have proved to be the methods with an additional diagnostic value, providing the physician with information about inflammatory, atrophic and metaplastic lesions of the stomach in dyspeptic *H. pylori *positive and negative patients. Bacteriological methods are suggested for detecting the putative antimicrobial resistance of *H. pylori*, aimed at successful eradication of infection in persistent peptic ulcer cases.

## Background

Treatment of peptic ulcer in accordance with relevant guidelines is becoming a common task for general practitioners [1-6]. In a post-treatment setting, in accordance with guidelines, prompt check-up of treatment results is recommended only in gastric ulcer cases with the use of ^13^*C-urea breath test *(^13^*C-UBT*) [2-5]. In a situation where patients have clinical symptoms after *H. pylori *eradication therapy, endoscopy is favoured in all peptic ulcer cases [6]. The aim of endoscopy is to establish the reason for clinical symptoms and to prove presence of peptic ulcer or malignancies, but also to support physicians and patients in the understanding of complaints [7]. Moreover, endoscopy allows determination of persistent *H. pylori *infection using endoscopy-based tests. Endoscopic biopsies alone are not considered adequate for confirming eradication of bacteria, although they might provide additional information about gastritis and dysplasia [8]. Use of more than one method in testing gastric specimens definitely enhances the diagnostic value when assessing the post-treatment *H. pylori *status [9].

Our aim was to assess the diagnostic value of different non-invasive (^13^*C-UBT*) and endoscopy-based diagnostic methods (visual endoscopy, classical cytological and histological examination of mucosal specimens, PCR and bacteriological methods) for monitoring patients after eradication therapy in a late post-treatment setting.

## Methods

### Patients

The study group was formed of 134 consecutive peptic ulcer outpatients who had been treated by 7-day triple therapy with metronidazole, amoxicillin and omeprazole in 1996. The group was observed at the outpatient department of Tartu University Hospital at 4 weeks, at 1 year (1997) and at 5 (2001) years after treatment [10]. Five years after treatment, 108 patients (81% of the initial group) were available for the follow-up of the clinical course of peptic ulcer. During the 5-year follow-up period only 11 (10 %) patients had relapses of peptic ulcer. For comparison of the diagnostic value of different diagnostic methods in a post-treatment setting, 34 patients were recruited. The inclusion criteria for this study group were resistant upper abdominal pain as the predominant complaint and compliance with all investigations (clinical symptoms, ^13^*C-UBT*, endoscopy, biopsy, bacteriology, PCR and cytology). The studied patients were not NSAID users.

### Methods

The patients passed the Gastrointestinal Symptoms Rating Scale (GSRS) test [11] in a validated Estonian translation. Dyspeptic syndrome (abdominal pain, heartburn, acid regurgitation, sucking sensation, nausea and vomiting) was registered on the 7-grade Likert scale for assessing severity of symptoms. The mean score of dyspeptic syndrome was calculated for each patient.

#### ^13^C-UBT

The subjects passed ^13^*C-UBT *drinking 100 mg ^13^*C*-urea; the test meal was citric acid and the time of specimen collection was 30 min. The test was provided, according to a standard protocol, from the Helsinki Keskuskatu Laboratory, Finland. The ratio of ^13^*CO*^2 ^to ^12^*CO*^2 ^in expired breath was measured by mass spectrometry and expressed in ml/mmol/kg (δ). An automated breath ^13^*C *analyser (ABCA) with chromatographic purification and a single inlet isotope ratio mass spectrometer (IRMS) were used. A difference of 5‰ in the content (δ^13^*C*) was considered positive for *H. pylori *infection.

#### Endoscopy of the upper gastrointestinal tract

The procedure was performed with the gastroscope Olympus-GIF 21. All mucosal defects were registered according to the Sydney classification for endoscopic evaluation [12]. Gastric ulcer was diagnosed if the ulcer was located at the angulus or above it. Duodenal ulcer was diagnosed if the ulcer was found in the duodenal bulb area.

#### Gastrobiopsy and histological examination

Five specimens from the antrum mucosa and five from the corpus mucosa were taken with medium-sized forceps. Two specimens were embedded in paraffin and the paraffin sections were stained using haematoxylin-eosin and Giemsa methods. The mucosal specimens were evaluated histologically according to the Sydney classification: presence of neutrophil infiltration, chronic lymphocytic inflammation, surface epithelial damage, atrophy, intestinal metaplasia, lymphoid follicles and *H. pylori *colonisation were evaluated on a three-grade scale both for the antrum and the corpus [12-14].

#### Bacteriological examination

One specimen from the antrum and one from the corpus were placed in the Stuart Transport Medium (Oxoid) and taken to the laboratory within two hours for bacteriological examination. The biopsy samples were homogenised with sterile glass powder and under a stream of CO_2 _and diluted in the Brucella broth (Oxoid). *H. pylori *was isolated on the Columbia Agar Base supplemented with 7% horse blood and 1% Vitox (Oxoid) or Isovitalex (BBL). The plates were incubated for 3–7 days at 37°C under microaerobic conditions (CampyBak, BBL or CampyGen, Oxoid). *H. pylori *was identified by Gram staining and by oxidase, catalase and urease reactions [15]. The sensitivity of the isolated *H. pylori *strains to clarithromycin was estimated by E-test. The antibiotic cut-off points employed for the E-test were 1.0 mg/l (NCCLS, 2002).

#### Cytological examination

One specimen was used for imprinting the cytology slides from the antrum and corpus mucosa, fixed with 96% ethanol and stained by Acridine Orange (Difco, BBL) [16]. The cytological specimens were studied under a fluorescence microscope (AXI Phot 2) where the morphotypes and the density of bacterial colonisation were evaluated [17]. A positive cytological diagnosis was based on the presence of typical helical *H. pylori *cells on the gastric mucosa and in the mucus layer.

#### PCR

For DNA extraction of *H. pylori *from a frozen gastric biopsy specimen, a previously described procedure was used [18]. The presence of the *glmM *gene in each strain was established by PCR using primers, the reaction mixture, and thermal cycling [19,20]. DNA from *H. pylori *NCTC 11637 (National Collection of Type Cultures, Central Public Health Laboratory, Colindale Ave., London NW9 5HT, England, United Kingdom) and the DNA-free reaction mixture were assayed in separate tubes in each PCR and were run as the positive and negative controls of the reaction, respectively. The PCR products were identified by electrophoresis on 2% agarose gels.

#### Criteria for evaluation

*H. pylori *was assessed positive if at least two tests were positive according to golden standard [21].

#### Statistical analysis

The data were analysed by Fisher's exact tests using the Jandel SigmaStat 2.0 program. Measurements from the GSRS were expressed as the mean values for dyspeptic syndrome.

### Ethics

The study was carried out in accordance with the Helsinki Declaration and was approved by the Ethics Committee of the University of Tartu.

## Results

Dyspeptic syndrome was found in all 34 cases. The mean GSRS score for the patients varied from 1.2 to 4.3.

The applied non-invasive test revealed *H. pylori *infection in half of the investigated patients: positive ^13^*C-UBT *was found in 17 out of 34 cases. There was no difference between the mean GSRS score values for the *H. pylori *positive and negative cases (2.8 ± 1.8 vs. 2.9 ± 1.7, p > 0.05).

On endoscopy, among the 34 patients, no ulcer or other mucosal defects were observed in 24 cases; erosions in the duodenal bulb were revealed in 6 cases and peptic ulcer was found in 4 cases (2 duodenal ulcers and 2 gastric ulcers). The data of *H. pylori *status and of the endoscopic finding are presented in Table 1.

**Table 1:** Comparison of the findings in *H. pylori *positive and negative cases in a late post-treatment setting

Patients (n = 34)		Non-invasive method	
		
		^13^*C-UBT *(+) n = 17	^13^*C-UBT *(-) n = 17
Invasive methods	Endoscopy: Normal	11	13
	Duodenal ulcer	2	0
	Gastric ulcer	2	0
	Erosions	2	4
	Cytology: *H. pylori *(+)	4*	Diverse forms of bacteria
	Histology: *H. pylori *(+)	17	0
	Bacteriology: *H. pylori *(+)	16	1
	PCR: *H. pylori *(+)	17	0

* typical morphology of *H. pylori *(the other cases showing diverse forms of bacteria)

A poor concordance was found between the visual examination of the gastric and duodenal mucosa on endoscopy and the applied non-invasive and invasive tests of *H. pylori *(accepting ^13^*C-UBT*, histological examination and PCR as the reference tests). The gastric and duodenal mucosa was visually normal in 11 *H. pylori *positive cases out of 17. On the contrary, only in 4 *H. pylori *positive cases did the endoscopic examination reveal the above mentioned peptic ulcers. Among the 6 cases with erosions of the duodenal mucosa, only two patients were *H. pylori *positive.

Comparison of the different diagnostic methods used for the detection of *H. pylori *is shown in Table 1. The results of ^13^*C-UBT *and PCR were consistent with the data of histological examination both in 17*H. pylori *positive and 17 negative cases. On bacteriological examination, only one case, which was *H. pylori *positive both by PCR and the histological tests, was *H. pylori *negative. In contrast, cytological examination assessed typical *H. pylori *bacterial cells in only 4 of the 17*H. pylori *positive cases (24%), while all other cases (both positive and negative for *H. pylori *by the other methods) displayed abundant bacteria of different morphotypes.

The data of the histological findings are presented in Table 2. Colonisation of the gastric mucosa by *H. pylori *was detected in 17 patients out of 34. Neutrophil infiltration, chronic inflammation, and surface epithelial damage both in the antrum and corpus mucosa were significantly expressed in the *H. pylori *positive cases (p < 0.001). Glandular atrophy and intestinal metaplasia were rarely observed both in the antrum and corpus mucosa of the *H. pylori *negative cases in comparison with the *H. pylori *positive cases, but the difference was not statistically significant (p > 0.05). Lymphoid follicles were more frequent in the antrum colonised with *H. pylori *(p < 0.05).

**Table 2:** Gastric mucosal findings (by the Sydney system) in *H. pylori *positive and negative cases

Gastric mucosal findings (Sydney system)	*H. pylori *(+) n = 17	*H. pylori *(-) n = 17	p values
Activity of neutrophil polymorphs			
Antrum	11/17	0/17	<0.001
Corpus	7/16	0/17	<0.05
Chronic inflammation			
Antrum	16/17	1/17	<0.001
Corpus	13/16	0/17	<0.001
Surface epithelial damage			
Antrum	13/17	0/17	<0.001
Corpus	8/16	0/17	<0.001
Glandular atrophy			
Antrum	7/17	2/17	NS*
Corpus	4/16	3/17	NS
Intestinal metaplasia			
Antrum	1/17	2/17	NS
Corpus	0/16	2/17	NS
Lymphoid follicles			
Antrum	6/17	0/17	<0.05
Corpus	5/16	2/17	NS

* NS, not significant (p > 0.05).

Bacteriological investigation revealed *H. pylori *in 16 biopsy samples of the antral mucosa, while highly (> 256 mg/l) clarithromycin resistant *H. pylori *strains were found in 3 cases.

## Discussion

Proper diagnostic and therapeutic management of patients with dyspeptic syndrome after *H. pylori *eradication therapy is of utmost importance for physicians as well for patients [7]. Several studies [22,23] have demonstrated the reliability of *H. pylori *tests used before treatment, while post-treatment testing is not yet adequately studied. However, in the case of long-lasting recurrent dyspepsia after *H. pylori *eradication therapy, endoscopy has been strongly recommended [4]. Our study shows that endoscopy gives useful information for the general practitioner both in the cases where peptic ulcer is found and in the cases where it is not found. In the case of a normal endoscopic finding, further management depends on the histological finding and on *H. pylori *status. Since persistent *H. pylori *positivity is always associated with possible peptic ulcer recurrence, the second line treatment according to bacterial susceptibility should be recommended. In the remaining cases where *H. pylori *is absent, the gastric mucosa is normal and no ulcer is detected, management of such patients should be aimed at establishment of other possible reasons for their complaints. Usually, a normal endoscopic finding reassures both the doctor and the patient [7].

A recent study of Ohkusa et al. [24] showed that even simple careful visual evaluation of the mucosa and the diagnoses of erythema and oedema correlated well with *H. pylori *infection. On the contrary, our results demonstrate that although all patients with recurrent peptic ulcer were *H. pylori *positive, the minor visual findings in the other cases were not in concordance with *H. pylori *colonisation. Usually, the mucosa was visually normal even when *H. pylori *was found, and, on the contrary, most duodenal erosions occurred in *H. pylori *negative patients. The clinical data of our patients did not suggest earlier use of NSAID, which would have been one of the main reasons for *H. pylori *negative erosions. Therefore, after treatment, in presence of complaints, it is important to obtain samples for the investigation of gastric mucosa specimens to enhance the value of endoscopic examination. We completely agree with the opinion that the value of using mucosal specimens for histological evaluation of late post-treatment *H. pylori *eradication is sometimes underestimated [9]. The non-invasive *H. pylori *test alone cannot solve the clinical problem of these patients. In our study, *H. pylori *negative patients had dyspeptic syndrome as well as gastric mucosal erosions, glandular atrophy and intestinal metaplasia. The last two lesions can presumably be associated with previous *H. pylori *infection and the follow-up of severe mucosal changes is recommended [25]. Hence it is evident that follow-up strategy should be considered also in *H. pylori *negative cases in accordance with endoscopic and histological findings.

Our study demonstrates that evaluation of the gastric mucosa with a focus on neutrophil and lymphocyte infiltration and epithelial damage is specific and sensitive for diagnosing *H. pylori *infection even after treatment, and that the diagnostic value of a histology-based decision is high. Today, the value of mucosal specimens for the post-treatment histological diagnosis of *H. pylori *is considered low assuming that *H. pylori *colonisation may be patchy, or coccoid forms are difficult to detect [25]. We have excluded patchy damage by using ^13^*C-UBT *in parallel with histological investigation.

Next, for detecting the coccoid forms of the bacteria, we used additionally PCR method. Our results show that the histological finding of *H. pylori *completely correlates with the results of ^13^*C-UBT *and PCR both in *H. pylori *positive and negative cases. This confirms the validity of the histological evaluation of mucosal specimens in the case of recurrent peptic ulcer or erosions. Moreover, in countries with a high rate of *H. pylori *infection and gastric cancer, it is especially important to follow up patients for detecting dysplasia and malignancies [26-29].

Surprisingly, brush cytology from the mucosa failed to detect *H. pylori *in cases where it was found by other methods. Cytology is highly evaluated for detection of *H. pylori *infection, as its agreement with histology is considered to be 100% [30]. Our results show that when patients had been treated with antibacterial drugs and still had dyspeptic complaints, cytological examination was not suitable for *H. pylori *detection, as different forms of the bacteria were found. The morphology of the helicobacters could have been modified for coccoid or otherwise non-typical forms. It is possible that some other bacteria might have colonised the mucosa due to reduced colonisation resistance after antibacterial treatment, failure of some intestinal functions or usage of medicines administered to relieve the feeling of discomfort [31-33].

Bacteriological investigation enabled to find a few clarithromycin resistant *H. pylori *strains, which may result in the failure of repeat triple therapy. As the macrolide clarithromycin is chemically stable and well tolerated [34], physicians often choose it for treatment of different infections. Therefore, if the physician plans to use macrolides, endoscopy and histological testing should be accompanied by bacteriological investigation. Regarding PCR, its main value, obtaining of fast results, is evidently not so important in post-treatment settings.

## Conclusions

In a late post-treatment setting, patients with dyspepsia should not be monitored only by non-invasive investigation methods; it is also justified to use the classical histological evaluation of *H. pylori *colonisation, PCR and bacteriology as they have shown good concordance with ^13^*C-UBT*. Moreover, endoscopy and histological investigation of a gastric biopsy have proved to be the methods with an additional diagnostic value, providing the physician with information about inflammatory, atrophic and metaplastic lesions of the stomach in dyspeptic *H. pylori *positive and negative patients. Bacteriological methods are suggested for detecting the putative antimicrobial resistance of *H. pylori*, aimed at successful eradication of infection in persistent peptic ulcer cases.

## Competing interests

The author(s) declare that they have no competing interests.

## Authors' contribution

HIM, IK and KLa carried out GSRS, endoscopy and gastrobiopsy. HK recruited patients, collected ^13^C-urea breath tests, and performed GSRS. KLõ carried out bacteriological examination. PH performed cytological examination. HA carried out molecular analysis and participated in the writing of the manuscript. HIM performed histological examination and statistical analysis, and participated in the design of the study and in the writing of the manuscript. MM coordinated the study and participated in the completion of the manuscript. All authors have read and approved the final version of the manuscript.

## Pre-publication history

The pre-publication history for this paper can be accessed here:



## References

[B1] Lee J, O'Morain C (1997). Who should be treated for Helicobacter pylori infection? A review of consensus conferences and guidelines. Gastroenterology.

[B2] Rubin G, Meineche-Schmidt V, Roberts A, de Wit N (2000). The use of consensus to develop guidelines for the management of Helicobacter pylori infection in primary care. European Society for Primary Care Gastroenterology. Fam Pract.

[B3] Roberts AP, Childs SM, Rubin G, de Wit NJ (2000). Tests for Helicobacter pylori infection: a critical appraisal from primary care. Fam Pract.

[B4] Malfertheiner P, Megraud F, O'Morain C, Hungin AP, Jones R, Axon A, Graham DY, Tytgat G (2002). Current concepts in the management of Helicobacter pylori infection--the Maastricht 2-2000 Consensus Report. Aliment Pharmacol Ther.

[B5] Opekun AR, Abdalla N, Sutton FM, Hammoud F, Kuo GM, Torres E, Steinbauer J, Graham DY (2002). Urea breath testing and analysis in the primary care office. J Fam Pract.

[B6] Vakil N, Hahn B, McSorley D (2000). Recurrent symptoms and gastro-oesophageal reflux disease in patients with duodenal ulcer treated for Helicobacter pylori infection. Aliment Pharmacol Ther.

[B7] Fennerty MB, Magaret N, Dalros L, Faigel D, Lieberman D, Shaw M (2001). Outcomes of Helicobacter pylori treatment in community practice and impact of therapeutic effectiveness information on physician behaviour. Aliment Pharmacol Ther.

[B8] Guidance for Industry. Evaluating Clinical Studies of Antimicrobials in the Division of Anti-Microbial Drug Products.. Helicobacter pylori.

[B9] Laine L, Sugg J, Suchower L, Neil G (2000). Endoscopic biopsy requirements for post-treatment diagnosis of Helicobacter pylori. Gastrointest Endosc.

[B10] Maaroos HI, Keevallik R, Kolk H, Kull I, Labotkin K, Tammur R (2001). Long-term endoscopic follow-up of peptic ulcer (PU): clinical course, endoscopic finding, state of antral and corpus mucosa and C13 breath test after H. pylori eradication. Gut.

[B11] Svedlund J, Sjodin I, Dotevall G (1988). GSRS--a clinical rating scale for gastrointestinal symptoms in patients with irritable bowel syndrome and peptic ulcer disease. Dig Dis Sci.

[B12] Tytgat GN (1991). The Sydney System: endoscopic division. Endoscopic appearances in gastritis/duodenitis. J Gastroenterol Hepatol.

[B13] Price AB (1991). The Sydney System: histological division. J Gastroenterol Hepatol.

[B14] Dixon MF, Genta RM, Yardley JH, Correa P (1997). Histological classification of gastritis and Helicobacter pylori infection: an agreement at last? The International Workshop on the Histopathology of Gastritis. Helicobacter.

[B15] Glupczynski Y, Megraud F and Lee A (1996). Culture of Helicobacter pylori from gastric biopsies and antimicrobial susceptibility testing. Helicobacter pylori: techniques for clinical diagnosis and basic research.

[B16] Chapin K, Murray PR, Baron EJ, Pfaller MA, Tenover FC and Yolken RH (1995). Clinical microscopy. Manual of clinical microbiology.

[B17] Bernhardt H, Knoke M, Weuffen W, Kramer A and Krasilnikov AP (1967). Der Magen-Darm-Tract. Handbuch der Antiseptic.

[B18] Sillakivi T, Aro H, Ustav M, Peetsalu M, Peetsalu A, Mikelsaar M (2001). Diversity of Helicobacter pylori genotypes among Estonian and Russian patients with perforated peptic ulcer, living in Southern Estonia. FEMS Microbiol Lett.

[B19] Bickley J, Owen RJ, Fraser AG, Pounder RE (1993). Evaluation of the polymerase chain reaction for detecting the urease C gene of Helicobacter pylori in gastric biopsy samples and dental plaque. J Med Microbiol.

[B20] Lu JJ, Perng CL, Shyu RY, Chen CH, Lou Q, Chong SKF, Lee CH (1999). Comparison of Five PCR Methods for Detection of Helicobacter pylori DNA in Gastric Tissues. J Clin Microbiol.

[B21] (1997). Technical annex: tests used to assess Helicobacter pylori infection. Working Party of the European Helicobacter pylori Study Group. Gut.

[B22] Leodolter A, Dominguez-Munoz JE, von Arnim U, Kahl S, Peitz U, Malfertheiner P (1999). Validity of a modified 13C-urea breath test for pre- and posttreatment diagnosis of Helicobacter pylori infection in the routine clinical setting. Am J Gastroenterol.

[B23] Senturk O, Canturk Z, Cetinarslan B, Ercin C, Hulagu S, Canturk NZ (2001). Prevalence and comparisons of five different diagnostic methods for Helicobacter pylori in diabetic patients. Endocr Res.

[B24] Ohkusa T, Fujiki K, Takashimizu I, Kumagai J, Tanizawa T, Eishi Y (2000). Endoscopic and histological comparison of nonulcer dyspepsia with and without Helicobacter pylori infection evaluated by the modified Sydney system. Am J Gastroenterol.

[B25] Sipponen P (2001). Update on the pathologic approach to the diagnosis of gastritis, gastric atrophy, and Helicobacter pylori and its sequelae. J Clin Gastroenterol.

[B26] Matysiak-Budnik T, Megraud F (1994). Helicobacter pylori in eastern European countries: what is the current status?. Gut.

[B27] Maaroos HI (1995). Helicobacter pylori infection in Estonian population: is it a health problem?. Ann Med.

[B28] Kolk H, Maaroos HI, Kull I, Labotkin K, Loivukene K, Mikelsaar M (2002). Open access endoscopy in an epidemiological situation of high prevalence of Helicobacter pylori infection: applicability of the guidelines of the European Society for Primary Care Gastroenterology. Fam Pract.

[B29] Bray F, Sankila R, Ferlay J, Parkin DM (2002). Estimates of cancer incidence and mortality in Europe in 1995. Eur J Cancer.

[B30] Cubukcu A, Gonullu NN, Ercin C, Alponat A, Kaur AC, Canturk Z, Paksoy N (2000). Imprint cytology in the diagnosis of Helicobacter pylori. Does imprinting damage the biopsy specimen?. Acta Cytol.

[B31] Loivukene K, Maaroos HI, Kolk H, Kull I, Labotkin K, Mikelsaar M (2002). Prevalence of antibiotic resistance of Helicobacter pylori isolates in Estonia during 1995-2000 in comparison to the consumption of antibiotics used in treatment regimens. Clin Microbiol Infect.

[B32] Nilius M, Strohle A, Bode G, Malfertheiner P (1993). Coccoid like forms (CLF) of Helicobacter pylori. Enzyme activity and antigenicity. Zentralbl Bakteriol.

[B33] Kusters JG, Gerrits MM, Van Strijp JA, Vandenbroucke-Grauls CM (1997). Coccoid forms of Helicobacter pylori are the morphologic manifestation of cell death. Infect Immun.

[B34] Alvarez-Elcoro S, Enzler MJ (1999). The macrolides: erythromycin, clarithromycin, and azithromycin. Mayo Clin Proc.

